# Supplementary material for: Wafer-Scale Epitaxial Low Density InAs/GaAs Quantum Dot for Single Photon Emitter in Three-Inch Substrate
Source: Nanomaterials (Basel). 2021 Apr 6;11(4):930. doi: 10.3390/nano11040930 (PMC8067461; doi:10.3390/nano11040930)
Supplement: Supplementary file 1 [file nanomaterials-11-00930-s001.pdf]

Supplemental material

I. RHEED patterns of evolution of GaAs surface during InAs deposition along [1-10] azimuth.

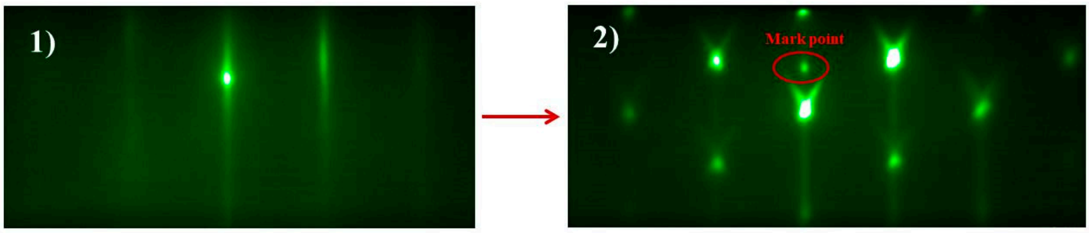

Figure S1. (1)–(2) surface reconstruction when InAs deposited on GaAs surface at the temperature of (Tc-25) °C along [1-10] azimuth observed by RHEED, streak 2×1 lines transfer to diffraction spots when reach critical thickness of θ_c, we marks the first diffraction point above 1_{st} Laue circle as the mark point as shown in (2)

II. AFM images of QD density change with different substrate temperature when deposit of 150%θ_c InAs.

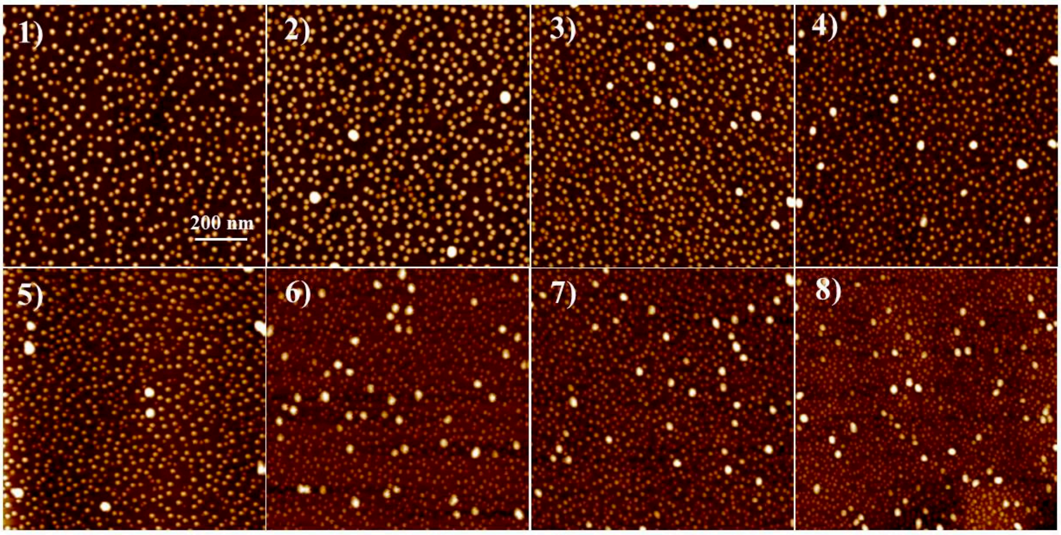

Figure S2. (1)–(8) 1 μm × 1 μm AFM images of eight other high density QD samples grown at different substrate temperature of 567 °C, 562 °C, 557 °C, 552 °C, 547 °C, 542 °C, 537 °C, 532 °C, correspond to density of $5.09 \times 10^{-6}/cm^2$, $5.63 \times 10^{-6}/cm^2$, $7.5 \times 10^{-6}/cm^2$, $8.94 \times 10^{-6}/cm^2$, $1.02 \times 10^{-5}/cm^2$, $1.32 \times 10^{-5}/cm^2$, $1.45 \times 10^{-5}/cm^2$, $1.72 \times 10^{-5}/cm^2$, respectively. Herein, the QD densities are calculated via the software of AFM Nanoscope Analysis. We note that the QD density increases almost linearly as a function of the deposition temperature.

III. AFM and height distribution of bimodal dots in 83%θ_C deposition

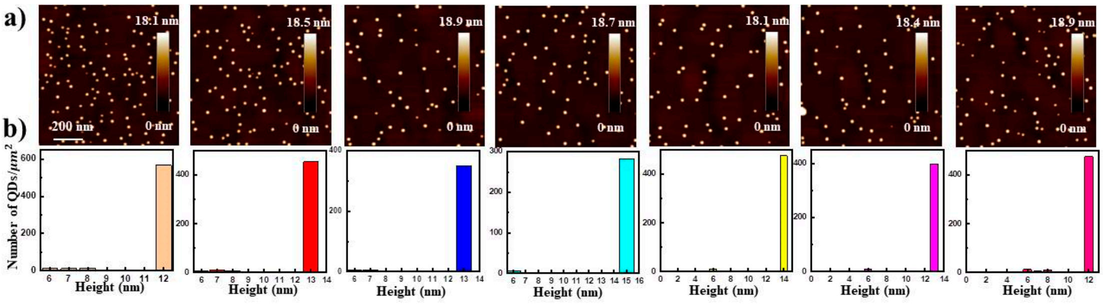

Figure S3. (a) Representative AFM images in 1 μm × 1 μm, (b) Statistical height distribution of QDs in 5 μm × 5 μm. The bimodal dots, with larger dots above 12 nm height and small dots below 8 nm height, mainly caused by different diffusion length of Indium atoms in different substrate.

IV. RHEED in situ monitoring and AFM images after 3 minutes In flushing

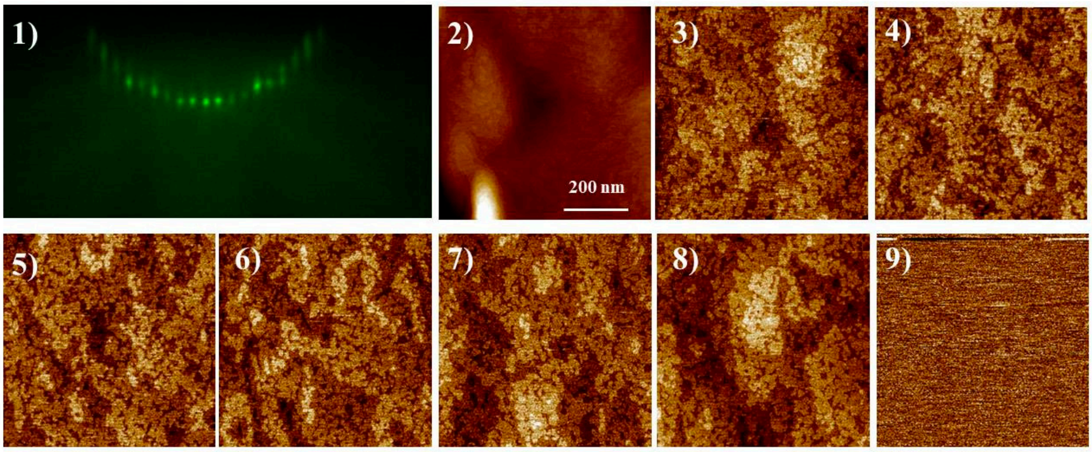

Figure S4. (1) 2×4 surface reconstruction after 3 minutes annealing under the temperature of 680 ℃ in situ observed by RHEED. We carried out an extra sample with the same experimental condition of sample E, which was stopped growth after the process of Indium flushing. As AFM images along [110] azimuth in (2)–(8) shown, the areal route mean square roughness $r_{(RMS)}$ is 0.723 nm, 0.19 nm, 0.165 nm, 0.169 nm, 0.177 nm, 0.196 nm, 0.217 nm, corresponding to the seven regions from −3 cm to +3 cm. The values of $r_{(RMS)}$ are nearly equal to that of the normal GaAs surface ($r_{(RMS)}$ of 0.14 nm in (9). The results of both RHEED and RMS indicate that the larger QDs are been flushed during the Indium flush process.
